# A review of selection criteria for ophthalmology training in the Western world

**DOI:** 10.1038/s41433-025-03850-x

**Published:** 2025-05-15

**Authors:** Thomas Muecke, Carson Petrash, Goran Petrovski, Stephen Bacchi, Robert Casson, Weng Onn Chan

**Affiliations:** 1 Health & Medical Science, University of Adelaide, Adelaide Health and Medical Sciences Building, North Terrace, Adelaide, SA 5000 Australia; 2 Ophthalmology Department, Royal Adelaide Hospital, Adelaide, 5000 Australia; 3 Ophthalmology Department, University Hospital, San Antonio, TX 78229 USA; 4 Ophthalmology Department, University Hospital, Ullevål, 0450 Oslo Norway; 5 Neurology Department, Flinders University, Bedford Park, SA 5042 Australia; 6 Neurology Department, Lyell McEwin Hospital, Elizabeth Vale, 5112 Australia; 7 Harvard Medical School, Harvard University, Boston, MA 02115 USA; 8 Massachusetts General Hospital, Boston, MA 02114 USA

**Keywords:** Education, Health occupations

## Abstract

**Background:**

To (a) analyse, compare and learn from the global variations in ophthalmology training applicant selection criteria, specifically CV assessment, and (b) provide a discussion of evidence supporting such selection criteria.

**Methods:**

An observational analysis on the selection criteria used to assess candidates applying to ophthalmology training programs within the US, Canada, European Union / European Economic Union (EU/EEA), United Kingdom (UK) and Australia and New Zealand (ANZ). Presence of a publicly available selection criteria policy for the 2025 intake was searched for on national and local college, society, federation and training program websites. The selection criteria employed for assessing applicant CV, and its associated scoring (if existent), were recorded for the included programs. Descriptive statistics was applied to these data.

**Results:**

174 accredited ophthalmology training programs were identified, and 51/174 publish a publicly available selection criteria policy. Overall, the most important criteria from ophthalmic training bodies in the Western world include research experience, academic achievements, particularly in the form of awards and prizes, references supporting evidence of favourable personal and professional characteristics, and evidence of involvement in extracurricular activities that produce evidence of a well-founded interest in ophthalmology.

**Conclusions:**

Each region adopts varying selection processes and frameworks, which, rather than reflect a standardised international approach to selecting an “ideal” ophthalmology trainee, perhaps select for the specific needs of the country and or training program. The study is limited by its observational nature.

## Introduction

Selection into ophthalmology training remains a challenge for applicants globally due to the intense competition for limited training positions. Assessment of ophthalmology training applicants typically begins with appraisal of the applicant’s curriculum vitae (CV). Information contained within the CV in combination with referee reports is commonly used to shortlist applicants for the next stage of assessment, which may include any combination of interviews, multiple mini-interviews, and/or situational judgement tests.

Globally, there are variations within the selection frameworks utilised for selecting applicants into ophthalmology specialty training [1]. University training programs in both the United States (US) and Canada develop their own selection frameworks locally. To align the needs of their program and local populus, the weighing of selection criteria is at the discretion of program directors at each university hospital training program; hence, the selection criteria vary between institutions [1]. Final year medical students apply to the National Medical Residency Match system, colloquially known as “The Match”. Designed by Nobel prize winner Alvin Roth to mimic the system he created to match kidney donors with recipients, “The Match” allocates applicants into specialty training programs (residencies) [1]. The US and Canada utilise San Francisco Match and Canadian Residency Matching System (CaRMS) respectively to streamline the matching service for training programs and applicants. In contrast, the selection process into ophthalmology training in Australia and New Zealand (ANZ) is standardised across the nations and coordinated by the Royal Australia and New Zealand College of Ophthalmologists (RANZCO), with successful applicants being matched into various training posts across the region. Prior to application, candidates must be medical officers of at least Post Graduate Year (PGY) 2-3, depending on the specialty. Alternatively, European countries match applicants into ophthalmology at local training institutions. Whilst multiple European ophthalmic governing bodies exist, including the of European Board of Ophthalmology (EBO) and the European Union of Medical Specialists (UEMS), they oversee and produce guidelines relevant to training rather than selection.

Whilst ophthalmic selection criteria used to assess an applicant’s CVs varies, it often involves ranking applicants across the following domains: (a) clinical research (b), clinical and ophthalmic experience (c) academic achievements (d) extracurricular activities and (e) diversity, equity and inclusion factors. The transparency of the CV assessment stage varies globally. For example, in Canada, selection criteria are published by each program utilising a standardised form on CaRMS. Contrarily, in the US, selection criteria may be published on residency training program websites. In ANZ, RANZCO publishes their publicly available standardised CV scoring criteria, identifying the relative point allocations towards different components within the CV. This allows for junior medical officers to understand the relative weighting of the various CV components, which ultimately reflects the specific needs of the specialty or health network. Resultingly, junior medical officers can work towards developing their CV components that are endorsed by the CV scoring criteria.

Utilising evidence-based approaches to developing ophthalmology training selection frameworks is paramount to selecting the optimal ophthalmologists of tomorrow. The objective of the present study is to (a) analyse, compare and learn from the global variations in ophthalmology training applicant selection criteria, specifically CV assessment, and (b) provide a discussion of evidence supporting such selection criteria.

## Methods

Ophthalmology residency/training programs within the US, Canada, European Union (EU) / European Economic Union (EEA), United Kingdom (UK), and ANZ were included, and were identified respectively through the Accreditation Council of Graduate Medical Education (ACGME) [2], Canadian Ophthalmic Society (COS) [3], EBO, National Healthcare System (NHS) [4] and the Royal Australia and New Zealand College of Ophthalmologists (RANZCO) [5]. Accredited training programs were included, and presence of a publicly available selection criteria policy for the 2025 intake was searched for on national and local college, society, federation and training program websites. The selection criteria employed for assessing applicant CV, and its associated scoring (if existent), were recorded for the included programs. Selection criteria related to intake of local medical graduates were included only, and criteria for international medical graduates was excluded. Descriptive statistics was applied to these data. Ethics approval was not required for the present study.

## Results

The number of accredited ophthalmology training programs identified were 174, and located in the US (126), Canada (15), EU/EEA (31), UK (1), and Australia / New Zealand (1). Of these programs, 51/174 publish a publicly available selection criteria policy, which identify which aspects of applicant CV are valued by the training program: US (33/126), Canada (15/15), EU/EEA (1/31), UK (1/1), and Australia / New Zealand (1/1). A summary of these 51 training programs selection criteria is in Table 1. Interestingly, only 3/51 programs that have publicly available selection policies, ANZ, the UK and Ireland, provide a CV scoring criteria, identifying the relative weighting of various criteria using point allocations.

**Table 1:** The proportions of ophthalmic selection policies who explicitly describe which selection criteria are valued, across training programs in the US, Canada, ANZ, and Europe.

Selection criteria	US programs (33)	Canadian programs (16)	Australia and New Zealand program (1)	European (EU/EEA) program: Ireland (1)	UK program (1)
**Research experience**	13/33	15/15	1/1	1/1	1
Publications	4/33	6/15	1	1/1	1/1
Presentations	3/33	2/15	1	1/1	1/1
Higher academic degrees	0/33	0/15	1	1/1	1/1
Research awards, grants and scholarships	0/33	1/15	1	1/1	1/1
**Rural exposure**	0/33	0/15	1/1	0/1	0/1
**Clinical experience**	16/33				
Evidence of surgical aptitude	0/33	5/15	0/1	0/1	1/1
Performance on clinical rotations	6/33	7/15	0/1	0/1	0/1
Medical student performance evaluation	Required on centralised application (SF Match)	Required on centralised application (CaRMS)	0/1	0/1	0/1
Reference letters from ophthalmologists	Required on centralised application (SF Match)	Required on centralised application (CaRMS)	Required on centralised application	1/1	Required on centralised application
Personal statement	Required on centralised application (SF Match)	Required on centralised application (CaRMS)	0/1	0/1	0.1
**Academic achievements**	22/33	15/15	1/1	1/1	1/1
Medical school performance (eg academic transcripts)	Required on centralised application (SF Match)	Required on centralised application (CaRMS)	0/1	1/1	0/1
Ophthalmology specific test	0/33	0/15	0/1	1/1 (Royal College of Ophthalmologists’ Duke Elder Undergraduate Prize Examination)	1/1 (Royal College of Ophthalmologists’ Duke Elder Undergraduate Prize Examination)
USMLE/COMLEX	Required on centralised application (SF Match)	0/15	0/1	0/1	0/1
Alpha Omega Alpha (AOA) membership	2/33	0/15	0/1	0/1	0/1
Prizes, awards and achievements	2/33	6/15	1/1	1/1	1/1
**Extracurricular activities**		15/15	1/1	1/1	1/1
Involvement in sports	0/33	3/15	Yes	0/31	0/1
Music	0/33	1/15	No	0/31	0/1
Volunteering	8/33	7/15	No	0/31	0/1
Leadership experience and positions	2/33	12/15	No	0/31	0/1
Teaching beyond the ward	3/33	2/15	No	0/31	1/1
Gaming	0/33	0/15	1/1	0/31	0/1
Participation in courses	0/33	0/15	0/1	1/31	1/1
Attendance at scientific conferences	0/33	0/15	0/1	0/31	1/1
Committee positions held	0/33	3/15	0/1	0/31	0/1
**Diversity, equity, and inclusion statement included in application**	11/33	7/15	1/1	0/1	0/1

## Discussion

Overall, the most important criteria from ophthalmic training bodies in the Western world include research experience, academic achievements, particularly in the form of awards and prizes, references supporting evidence of favourable personal and professional characteristics, and evidence of involvement in extracurricular activities that produce evidence of a well-founded interest in ophthalmology. The US and Canada reportedly place a universally greater weighting on previous medical school experience through assessing transcripts [6]. Contrarily, the ANZ program currently place 57.6% of their point allocation within their CV scoring criteria towards diversity, equity, and inclusion factors [7]. Whilst programs may still score and place value on criteria not described in their selection policy, which limits our data, it is important to consider the impact of lack of selection transparency. Allowing applicants to understand what is required of them to successfully enter a specialty career outside of “word of mouth” approaches, allows them to develop their experiences during and after medical school accordingly. This transparent approach to selection can be seen in ANZ and Ireland, the only two programs globally who publish scoring criteria within their selection policy.

It is important to recognise that other than Ireland, no ophthalmology training programs in the EU/EEA provide selection policies. Organisations including the EBO and UEMS aim to standardise ophthalmology training. Whilst they generally do not account for selection policies, the UEMS charter states that selection processes should be “transparent” and utilise “established selection procedures”. Ultimately, however, each European country determines whether they adopt recommendations and guidelines from these bodies[8]. Previous work calls for further standardisation of ophthalmology training across Europe and recommends standardised accreditation rules[8, 9]. Our findings support this work by providing evidence that standardised selection into ophthalmology is lacking. Further standardisation may improve ophthalmic outcomes, resulting in increased skills interchange and collaboration across Europe and the global ophthalmic community.

It is important to further delve into the evidence supporting the scoring of varying applicant assessment domains, such that training programs can develop ophthalmic selection criteria accordingly. As a result, they can produce and publish criteria that is (a) transparent and readily available to applicants, (b) in line with the current evidence, and (c) meets the needs of the local region.

### Research involvement

Previous applicant research experience is valued in a majority of the 51 programs that publish a publicly available selection policy: 13/33 US, 15/15 Canadian, and the ANZ, UK and Ireland programs. However, specific reference to publications and presentations is made only in 4/13 and 3/13 US and 6/15 and 2/15 Canadian programs respectively. Transparent reference to these activities would allow applicants to fully understand what is valued as “research experience” and to develop their CV accordingly. Furthermore, highlighting the importance of various clinical research activities in selection policies may allow for selection of applicants who are likely to engage in evidence-based medicine and continue contributing academically during and after their training [10]. Additionally, it may facilitate the generation of novel insights to improve ophthalmic practice. Interestingly, these data suggest that attainment of higher academic degrees is not universally valued by US and Canadian programs. Similarly, research awards, grants and scholarships are mentioned in only 1/15 Canadian programs. Contrarily, the ANZ, UK, and Ireland directly mention the importance of all identified aspects of research involvement.

Emerging evidence suggests ophthalmic trainee applicant features that may predict future research productivity. These include tier of US medical school attended, pre-training research involvement including attainment of a higher degree by research and publication of clinical research [11–13]. It is important to recognise that these data are specific to the US, and as a result the generalisability of these findings to other countries is unknown [14]. Further studies are required to improve the robustness of this finding, and additional analysis of other selection criteria and its prediction of research productivity could be of interest.

Clinical research involvement also suggests valued applicant attributes including teamwork abilities, critical thinking, and motivation. Furthermore, academic commitment through higher degrees by research may reflect a longstanding interest and engagement in ophthalmology [15]. However, weighting clinical research for applicant selection poses several limitations. One such limitation is the non-uniform nature of research availability and accessibility, particularly for underrepresented minorities [16], and those studying or training outside of academic centres. Furthermore, metrics including publication and citation count, which are typically used to assess candidates research experience, may provide a limited perspective on applicant research output [17]. As a result, conducting holistic analyses of the applicant’s author level publication metrics, and placing greater value on research achievements and higher degrees, may be a more useful way of utilising research experience to rank ophthalmic trainee applicants [17].

### Previous ophthalmic experience

Interestingly, previous ophthalmic clinical experience and evaluations, and examinations are undervalued in the included ophthalmic applicant selection policies. It is important to consider the importance of selecting applicants for each of these traits and the evidence that surrounds such selection criteria. The value placed on previous ophthalmic and clinical experience is disproportionately weighted between regions. Whilst all programs require reference reports from ophthalmologists in the selection of applicants, implying previous ophthalmic experience, this selection modality is vulnerable to operator and selection bias. Interestingly, previous ophthalmic and clinical experience is disproportionally weighted, as only 6/33 and 7/15 US and Canadian programs mention value for clinical evaluations. The UK program provides scoring to previous ophthalmic attachments, informal attendance in ophthalmic clinics and theatres, and evidence of training on the ophthalmic microsurgical simulator (Eyesi). Furthermore, the ANZ and Ireland programs do not require previous ophthalmic experience. The ANZ program, however, makes mention that previous experience may be used to weight program allocation once approved into ophthalmology training centrally.

Previous involvement in ophthalmology may be an important criterion to weight applicants. Evidence shows that prior experience within a specialty may also reduce rates of dropout [18], and may lead to most cost-effective clinical practice [19]. A high weighting towards clinical experience may incentivise potential applicants to seek out more opportunities in the relevant fields. This may result in applicants who are “ready to go”, having good core ophthalmic skills necessary to facilitate early training. Whilst prior clinical experience in the specialty of interest demonstrates an applicant’s keenness and interest, it also demonstrates that the individual has had a practical opportunity to develop a clear understanding of the specialty’s demands, challenges, and patient population. Further, it provides applicants time to develop and refine clinical skills relevant to the specialty. Weighting applicants prevocational non-ophthalmic clinical experience may also be of value. Such comprehensive experiences may provide opportunities for applicants to develop a wider range of personal and professional skills, and multidisciplinary relations. This will allow them to thrive in future ophthalmic workplaces and further aid the healthcare system [20]. Evidence demonstrates that prevocational general clinical training may enhance future performance during and after specialty training [21].

Our findings reveal that numerous training programs weigh CV elements of rural exposure, research, and diversity higher than clinical experience, suggesting that the relevant surgical and clinical skills may be learnt during the training program itself. It may be of value to consider utilising other methods of assessing ophthalmology experience. This includes considering applicant microsurgical aptitudes and technical ability, as endorsed by 5/15 Canadian programs. Emerging evidence suggests, however, that such aptitudes can be indirectly selected for in ophthalmic applicants by scoring for criteria including gaming [22–25]. Interestingly, ANZ is the only program that scores for gaming. Furthermore, utilisation of modalities including ophthalmology anatomy demonstration teaching may be of benefit, as it provides an opportunity to develop clinical anatomy knowledge, teaching, and scholar skills, whilst simultaneously further developing “out-of-the-textbook” clinical skills. Studies are warranted that explore an applicant’s previous prevocational ophthalmic experiences and their future ophthalmic clinical and surgical technical performance.

### Academic achievements

Only US and Canadian programs require academic transcripts in selection. In the UK, the Royal College of Ophthalmologists sponsor an ophthalmology examination named the Duke Elder Undergraduate Prize Examination. This examination, which is also recognised in the Irish ophthalmic training program, provides an optional opportunity for candidates to demonstrate their ophthalmic knowledge and understanding. Furthermore, high scoring candidates may be provided with additional points towards ranking. For example, those who score in the top 10% will receive two additional points towards scoring and special commendation. Only 2/33 and 6/15 US and Canadian programs respectively, and the ANZ, UK and Ireland programs explicitly value academic achievements, prizes and awards.

Attainment of prior academic achievements suggests that the applicant’s talent and potential to be able to fulfil the requirements of completing an ophthalmology training and accreditation examinations. There exists evidence to suggest that applicants who have USMLE Step 1 and 2 [26–28], medical school honours [28], high clerkship grades [28] AOA membership [28] are predicted to perform better during and after ophthalmology training. Interestingly, attainment of a PhD [28] previous completion of clinical research [28], and medical school GPA [27] was not shown to be performance predictive. Outside of ophthalmology, correlations between students’ performance in medical school and their achievements as a junior doctor has been demonstrated [5].

Programs who value only academic achievement in the form of medical school performance may limit their selection. Applicants with a broad range of academic achievements, through areas such as university accolades or competitive scholarships, demonstrate their ability to navigate substantial workloads, effectively allocate their time and resources, and be a potential future leader. This is particularly important in the context of the reported elevated rates of burnout in surgical specialties [29]. It is important to consider the limitation of scoring for academic achievements outside of medical school and examination-based performances. An applicant’s capacity to garner academic prizes, achievements and awards may be partly dictated by the institution they attend.

### Extracurricular activities

Extracurricular activities provide an opportunity for applicants to indirectly portray their non-cognitive and soft skills and traits developed within and outside of an ophthalmic environment, including motivation, dedication and teamwork. However, extracurricular activities are not consistently weighted across global training programs. Involvement in music is only valued by 1/15 Canadian programs. Similarly, sports are only valued by 3/15 Canadian programs and the ANZ. Interestingly, the ANZ only provides scoring towards sports when it is accompanied by achievement at a national or international level. This is important to consider as such an accomplishment is time and resource intensive, and its non-uniform accessibility may leave this criterion unattainable to many applicants. Teaching is valued by 3/33 US and 2/15 Canadian programs. Additionally, 2/33 US and 12/15 Canadian programs mention leadership experience and positions, however, do not elaborate further. Weighting applicants for extracurricular activities also provide an opportunity for training programs to expect a deeper level of commitment of the applicant to ophthalmology, through participation at ophthalmic conferences, completion of skills courses and from holding positions in committees. However, only the Ireland program values completion of courses, and only 3/15 Canadian programs weight for holding committee positions.

Mounting evidence reports that extracurricular activities including sport, music, teaching and volunteering may predict valuable traits and characteristics of individuals. For example, excellence in team sports predicts academic success and professional resilience as a resident [30, 31]. Furthermore, evidence suggest a positive correlation may exist between volunteering and annual grade point average (GPA) [32, 33]. Whilst evidence is not directly medically or ophthalmic related, it is important to consider applicant’s “soft” and non-cognitive skills for the extent they may indirectly improve academic performance. For example, involvement of medical students in teaching is reported to help develop soft skills including leadership, communication and time management [34, 35]. Furthermore, an umbrella review reveals that volunteering improves empathy, leadership and teamwork skills [36]. Similarly, involvement in musical pursuits suggests enhancements in cognitive skills such as working memory [37, 38]. Finally, it is globally recognised that teamwork and professional relations are essential in constructing a more effective healthcare delivery system.

### Diversity, equity and inclusion (DEI)

Based on US survey data, ophthalmology is reported to be less diverse in regard to race, ethnicity, and gender when compared to the general populus, medical students, and other medical specialties [39]. Whilst population data regarding gender within ophthalmology is well described, data are lacking in regard to ophthalmic diversity in sexual identity, socioeconomic status and disability [39]. Furthermore, recent evidence suggests that the diversity of ophthalmologists in the US is not in keeping with the rising rates of those racial and ethnics groups who are significantly less represented in medicine than in the general population [39]. As a result, with absent change, rates of eye impairment in these underrepresented groups may continue to increase [39]. The engagement of DEI populations in ophthalmology selection varies across the included countries: 11/33 and 7/15 US and Canadian programs respectively, include a statement regarding the engagement of DEI populations to apply for their training program in the application portal, or in an easily accessible location on their residency websites. Whilst Ireland and UK do not include such statement, the ANZ ophthalmology program provides scoring to improve the selection of DEI populations. This particularly includes rural and remote, and First Nations identifying individuals.

Efforts to include DEI factors in selection promote representation of all genders, races, ethnicities, sexual representations, socioeconomic status, ages, and disability levels in ophthalmology [7, 40]. As a result, this facilitates engagement of a wider proportion of the populus in eye care. Subsequently, optimal care can be provided to minorities of the community. Engagement of rural and remote communities is particularly important in Australia [41], where 28% of the populus live in rural and remote regions [42]. To further promote DEI efforts in ophthalmology globally, selection can include valuing those who have completed cultural competency training and been involved in DEI advocacy programs and initiatives.

## Conclusions

The findings of the present study indicate that throughout the Western world, there are variations in selection criteria used to initially assess and shortlist candidates for ophthalmology training program interviews. Each region adopts varying selection processes and frameworks, which, rather than reflect a standardised international approach to selecting an “ideal” ophthalmology trainee, perhaps select for the specific needs of the country and or training program. Our findings and learnings from analysing ophthalmic training program selection policies suggests that applicants may be selected based on criteria within the following three domains: (a) activities and achievements that suggest the applicant’s cognitive abilities: research output and influence, academic achievements, performance on clinical attachments, (b) activities that reflect applicant’s non-cognitive factors (e.g. professionalism, teamwork skills, dedication): extracurricular involvement, teamwork abilities in research and clinical engagements based off of evaluations and subjective matters, (c) diversity, equity and inclusion: increasing the diversity of trainees and specialists so that a greater proportion of the populus can be engaged. Publishing publicly available selection policies provides transparency for applicants regarding what is required for a competitive application and the overall values and requirements of the training program and its local region. It should be noted that while transparency is important, in some ways it can be self-defeating. Programs want to attract applicants whose interests are genuine and inspired without regard to the short-sighted goal of matching. Applicants who view research or scholarly pursuit as a means to an end will likely leave those activities after having matched. Publishing the selection criteria could lead to a “check-box” approach for applicants, rather than fostering a culture of sustained commitment and meaningful contribution. The study is limited by its observational nature. Future research could assess the correlation between ophthalmic training program selection criteria and future performance during and after training.

## Summary

### What was known before

- Different countries throughout the western world utilise varying frameworks to select applicants into ophthalmology training programs.
- The evidence base surrounding applicant selection into ophthalmology training programs is limited across the western world.
- Utilising evidence-based approaches to developing ophthalmology training selection frameworks is paramount to selecting the optimal ophthalmologists of tomorrow.

### What this study adds

- The majority of ophthalmology training programs in the western world do not publish publicly available applicant selection policies.
- Overall, the most important criteria from ophthalmic training bodies in the Western world include research experience, academic achievements, particularly in the form of awards and prizes, references supporting evidence of favourable personal and professional characteristics, and evidence of involvement in extracurricular activities that produce evidence of a well-founded interest in ophthalmology.
- Utilisation of standardised and evidence-based applicant selection tools across the western world will enhance international ophthalmic collaboration.

## Data Availability

The datasets generated during and/or analysed during the current study are available from the corresponding author on reasonable request.
